# Socio-Demographic, Environmental, and Clinical Factors Influencing Osteoporosis Control in Community Pharmacies of Lahore Pakistan

**DOI:** 10.3390/healthcare13243291

**Published:** 2025-12-15

**Authors:** Muhammad Zahid Iqbal, Aqsa Malik, Naeem Mubarak, Tahneem Yaseen, Seerat Shahzad, Khalid M. Orayj, Saad S. Alqahtani

**Affiliations:** 1 Department of Clinical Pharmacy, College of Pharmacy, King Khalid University, Abha 61421, Saudi Arabia; korayg@kku.edu.sa (K.M.O.); ss.alqahtani@kku.edu.sa (S.S.A.); 2 Department of Pharmacy Practice, Faculty of Pharmaceutical Sciences, Lahore University of Biological & Applied Sciences, Lahore 53400, Pakistan; imaqsaaa461@gmail.com (A.M.); naeem.mubarak@ubas.edu.pk (N.M.); tahneemyaseen@gmail.com (T.Y.); seeratshahzad3@gmail.com (S.S.)

**Keywords:** osteoporosis, community pharmacy, Lahore, Pakistan, vitamin D, sunlight exposure, glucocorticoids, diabetes, chronic kidney disease, rheumatoid arthritis, smoking, bone mineral density

## Abstract

**Highlights:**

**What are the main findings?**

- Diabetes, glucocorticoid exposure, smoking, and limited sunlight exposure were the strongest factors associated with high fracture risk among adults screened in community pharmacies in Lahore, Pakistan.
- Most socio-demographic characteristics showed minimal independent influence on osteoporosis fracture risk after multivariable adjustment.

**What are the implications of the main findings?**

- Community pharmacies can serve as effective sites for early identification of individuals at high risk of osteoporotic fractures.
- Targeted pharmacist-led screening and counseling may support timely referral for bone mineral density testing and improve osteoporosis management in outpatient settings.

**Abstract:**

**Background and Objectives**: Osteoporosis risk in real-world, outpatient settings is shaped by intersecting socio-demographic, environmental, and clinical factors. We evaluated predictors of fracture risk status among adults seeking care in community pharmacies in Lahore, Pakistan. **Materials and Methods**: We conducted a cross-sectional study across urban and suburban pharmacies using a validated questionnaire aligned with international guidelines. Participants were classified as lower risk (osteopenia/osteoporosis without fragility fracture) or high risk (≥1 fragility fracture with clinical osteoporosis). Associations between candidate factors and risk status were examined using univariate and multivariable logistic regression analyses. **Results**: Of 286 participants, 53.1% were classified as lower risk. After adjustment, most sociodemographic characteristics were not independently associated with fracture risk status, except monthly income. Strong associations were observed for diabetes (AOR = 0.005, 95% CI 0.0007–0.040; *p* < 0.001), short-term glucocorticoid use (AOR = 32.33; *p* = 0.004), current smoking (AOR = 14.23; *p* = 0.002), ex-smoking (AOR = 4.95; *p* = 0.042), and lack of sunlight exposure (AOR = 7.09; *p* = 0.019). CKD, rheumatoid arthritis, and vitamin D insufficiency demonstrated borderline non-significant trends. Multivariable modeling did not include “not tested” categories or sparse variables. **Conclusions**: In Lahore’s community pharmacies, diabetes, CKD, RA, glucocorticoid exposure, smoking, and sunlight/vitamin D-related factors were the dominant correlates of osteoporosis fracture risk status, whereas most socio-demographic factors exerted limited independent effects. Pharmacy-anchored screening and counseling focused on these high-yield clinical indicators alongside timely BMD referral and guideline-concordant therapy may help identify individuals at elevated fracture risk.

## 1. Introduction

Osteoporosis is characterized by reduced bone mass, damage to bone tissue, and changes in the bone’s microstructure [1]. These alterations reduce bone strength and make fractures more likely [2]. Hip fractures are the most devastating fractures in terms of morbidity, death, and medical expenses [3]. Osteoporosis is more prevalent among Caucasians, women, and the elderly [4].

Globally, one in every three women and one in every five men over the age of 50 suffer from osteoporosis [5]. In both Europe and the United States, it is projected that 40% of post-menopausal women and 30% of males will have an osteoporotic fracture in their remaining lifetime [4]. Available data show that the problem of osteoporosis is serious in Pakistan, with rates ranging from 5.6–17.8% in premenopausal women and 20–49.3% in postmenopausal women [6]. More Pakistani women will experience fractures linked to osteoporosis in the future, which will lower their quality of life [7].

Since osteoporosis only manifests as fractures, risk assessment is required to determine who is more likely to experience clinical events [8]. Therefore, using a test called Dual energy X-ray absorptiometry (DEXA) is important for early detection and to help prevent fractures [9]. The National Osteoporosis Foundation recommend that all women over the age of 65, men over the age of 70, postmenopausal women with bone loss due to medical reason, aged 50 and older with additional risk factors of fragility fractures be screened for osteoporosis [10].

Osteoporosis is generally classified into two major types: primary osteoporosis and secondary osteoporosis [4]. Primary osteoporosis occurs frequently in postmenopausal women and those over the age of 70 [11]. Secondary osteoporosis is caused by underlying medical conditions, systemic illnesses, hormonal problems, malignancies, prolonged drug usage, and lifestyle-related factors, including depression. In most cases, secondary osteoporosis is multifactorial and is often diagnosed after an atraumatic fracture [12].

Key modifiable factors that contribute to osteoporosis include cigarette smoking, low body mass index (BMI), Vitamin D deficiency, excessive alcohol consumption, psychological stress, unintended weight loss, physical inactivity or fall risk [13]. In contrast, non-modifiable factors include increasing age, female gender, family history, a history of fractures in adulthood and dementia [14]. Moreover, several studies further highlight that depression may elevate the risk of osteoporosis [15]. Type 2 diabetes is connected with an increased incidence of osteoporosis, particularly among non-elderly individuals, regardless of gender [16].

Over 95% of people with osteoporosis have one or more concomitant disorders, including increased risk of arthrosis, arthritis, chronic low back pain, depression, and chronic heart failure [17]. Nonpharmacological therapy of osteoporosis involves appropriate vitamin D and calcium intake, quitting smoking, weight-bearing physical exercise, limiting alcohol and caffeine use, and fall-prevention strategies [18]. Adequate vitamin D and calcium consumption helps prevent and treat vitamin D deficiency-related issues such as osteoporosis and fractures [19].

Present treatments for osteoporosis include drugs that promote bone formation, suppress bone resorption, or combine both effects [20]. Treatment options include estrogen therapy, calcitonin, selective estrogen receptor modulator (SERM), four bisphosphonates, monoclonal antibody therapies, parathyroid hormone analog teriparatide, new drugs like abaloparatide and romosozumab, and cathepsin K inhibitor odanacatib [21]. Community pharmacists may help manage chronic diseases in a variety of ways [22]. They are transforming from basic dispensing to in-person services like disease prevention, health promotion, immunization, emergency contraception, medication counseling, and disease monitoring [23].

Only a few studies have examined regarding how socio-demographic, environmental and clinical factors collectively affect osteoporosis management, and this gap is more evident in middle-income countries like Pakistan. In response to increasing disease burden and importance of understanding disease management outside hospital environments, this study aims to address the effect of multiple factors influencing patients seeking services at clinical pharmacies in Lahore. By focusing on an outpatient population, this research offers broader perspective on osteoporosis treatment and suggest community-level approaches to improve disease management in the real world.

## 2. Methodology

A cross-sectional observational study was conducted to assess the sociodemographic, environmental, and clinical factors influencing osteoporosis management among patients visiting community pharmacies in Lahore, Pakistan. Lahore was chosen in particular because of its socioeconomically diversified population. Numerous urban and suburban areas were included in the study to ensure demographic diversity and improve the findings’ generalizability.

Medical care standards and recognized international recommendations, particularly the National Institute for Health and Care Excellence (NICE) guidelines, were used to develop a validated structured questionnaire. The questionnaire was developed based on international osteoporosis guidelines and reviewed by a panel of experts in clinical pharmacy and osteoporosis for content validity. A pilot test was conducted with 20 participants to ensure clarity and relevance of items. However, full psychometric validation using Classical Test Theory or Item Response Theory was not performed, and the instrument should therefore be considered content-validated but not fully psychometrically validated. The survey was divided into two major sections. The first section gathered extensive sociodemographic information such as age, gender, marital status, employment, income level, level of education, and urban or rural residence. The second portion focused on clinical, environmental, and lifestyle variables that impact osteoporosis. These factors include clinical factors such as menopausal status, comorbidities (diabetes, RA, CKD), history of fragility fractures, bone mineral density, vitamin D level and the use of long-term medications (glucocorticoids). Other environmental/lifestyle factors include sunlight exposure, vitamin D and calcium diet, physical activity, smoking status, alcohol consumption and BMI.

For the comparative analysis of factors influencing osteoporosis management, study participants were stratified into two groups based on their osteoporosis control status. The classification criteria incorporated two key clinical parameters: history of fragility fractures and bone mineral density (BMD) assessment. Participants were stratified into two clinically relevant categories based on fracture risk status, consistent with National Osteoporosis Foundation (NOF) and NOGG guidelines:

- High-Risk Group: Individuals with ≥1 low-trauma (fragility) fracture, regardless of BMD score.
- Lower-Risk Group: Individuals with osteopenia or osteoporosis (based on BMD) but without any history of fragility fracture.

Fragility fracture is widely recognized as the strongest clinical indicator of future fracture risk and is considered a high-risk state requiring targeted management. Thus, this classification reflects fracture risk stratification rather than “disease control”. This operational definition emphasizes fracture occurrence as a critical indicator of poor disease control, consistent with the primary therapeutic objective of osteoporosis management namely, the prevention of fractures.

The study primarily focused on three regions: Gulberg, Garhi Shahu, and Harbanspura. Prior to data collection, pharmacists in charge called and addressed pharmacies in these locations to obtain written approval. Patients were chosen using convenience sample methods, with a focus on those who visited community pharmacies for osteoporosis prescription medicines or associated counseling. Participants were informed of the study’s purpose, and their anonymity was assured. Participation required written informed permission, which was obtained from each participant after explaining the study’s aims and methodologies.

A total of 286 osteoporotic people volunteered to participate in the research. To confirm the data’s completeness and accuracy, in-person structured interviews were conducted in each pharmacy. To eliminate interviewer bias, interviews were carried out by certified researchers. Throughout the study, participant confidentiality and anonymity were strictly protected. 

The Research and Ethical Review Committee of Lahore University of Biological and Applied Sciences authorized this work. The committee assessed and approved the study protocol, which was awarded ethical approval under reference number MZI/56/23. The study maintained participant autonomy and anonymity by strictly following the ethics committee’s norms and regulations.

## 3. Statistical Analysis

The data were analyzed with the Statistical Package for Social Sciences (SPSS) 26.0. (Armonk, NY, USA) Patients’ characteristics and confounding variables were described using descriptive statistics. Analytical statistical methods were also used to determine the association between sociodemographic, environmental, and clinical variables and osteoporosis control outcomes. The adequacy of the sample size for logistic regression was evaluated using the Events-Per-Variable (EPV) approach. With 133 individuals in the high-risk (fracture-present) category and a maximum of 10 predictors included in the multivariable logistic regression model, the EPV was approximately 13.3, exceeding the recommended minimum of 10 EPV required for reliable parameter estimation. Therefore, the achieved sample size of 286 participants provided sufficient statistical power for the regression analyses conducted. Initial associations between categorical predictors and fracture risk status were assessed descriptively. Inferential analysis was performed using univariate logistic regression followed by multivariable binomial logistic regression, with *p* < 0.05 considered statistically significant. Univariate screening was conducted using simple logistic regression, followed by multivariable binomial logistic regression to identify independent predictors. Chi-square tests were used only for descriptive comparison, not for inferential modeling.

## 4. Results

A total of 286 participants were enrolled. Most were female (63.3%), and older adults predominated (41–65 years: 37.8%; >65 years: 36.4%). Nearly 36.7% were retired and 35.5% had no formal education. Residence was evenly split (urban: 51.0%; rural: 49.0%). Over half lived in rented housing (57.7%) and in joint-family settings (61.9%). Monthly income was <20,000 PKR (18.2%), 20k–50k (33.9%), 50k–100k (26.2%), and >100k (21.7%). Clinically, menopause was reported by 54.5% of women, fragility fracture by 46.5%, CKD by 37.8%, diabetes by 52.1%, and rheumatoid arthritis by 23.1% (with 27.3% not tested). Family history of osteoporosis was reported by 39.9%, and long-term glucocorticoid use by 21.3%. For behaviors/exposures, current smoking was reported by 40.2%, physical activity ≥150 min/week by 56.3%, sunlight exposure by 61.2%, and adequate vitamin D/calcium intake by 52.1%. Regarding BMD, osteoporosis was reported by 27.6%, osteopenia by 11.5%, and normal density by 48.6%. Alcohol use was reported by 14.0% participants. More details can be found in Table 1.

On univariate analysis, age > 65 years had lower odds of control than 18–40 years (OR 0.54, 95% CI 0.30–0.99; *p* = 0.046), but this association attenuated after multivariable adjustment (AOR = 0.59, 95% CI 0.30–1.15; *p* = 0.120). Other socio-demographic factors—including gender, occupation, education, marital status, residence, and living/family conditions—were not independently associated with control after adjustment. Monthly income showed a distinct pattern: compared with <20,000 PKR, the 20k–50k group had higher adjusted odds of control (AOR = 2.98, 95% CI 1.27–7.03; *p* = 0.012), whereas the >100k group was associated with lower adjusted odds (AOR = 0.37, 95% CI 0.17–0.80; *p* = 0.012). Furthermore, details can be obtained in Table 1.

Table 2 and Table 3 show that several clinical and environmental factors were significantly associated with high-risk osteoporosis status. In the adjusted analysis, individuals without diabetes had markedly lower odds of being in the high-risk group (AOR = 0.005, 95% CI 0.0007–0.040; *p* < 0.001). Short-term glucocorticoid use was also independently associated with higher odds of high-risk status (AOR = 32.33, 95% CI 3.05–342.66; *p* = 0.004), whereas long-term use did not reach statistical significance. Current smoking showed a strong association with high-risk status (AOR = 14.23, 95% CI 2.66–76.04; *p* = 0.002), and ex-smoking also demonstrated a significant association (AOR = 4.95, 95% CI 1.06–23.11; *p* = 0.042). Lack of sunlight exposure remained a significant predictor (AOR = 7.09, 95% CI 1.38–36.40; *p* = 0.019).

Other clinical variables, including chronic kidney disease, rheumatoid arthritis, and vitamin D insufficiency, exhibited trends toward association but were not statistically significant after adjustment. Variables such as alcohol consumption, dietary intake, physical activity, BMI, and “not tested” categories were excluded from the adjusted model due to sparse cell counts or lack of statistical stability. Overall, the adjusted findings highlight diabetes, short-term glucocorticoid exposure, smoking status, and sunlight exposure as the most robust correlates of high-risk osteoporosis status in this cohort.

## 5. Discussion

Among the 286 adults visiting community pharmacies in Lahore, slightly more than half (53.1%) fell into the lower-risk category, indicating that fragility-related risk factors remain notably prevalent in this population. After multivariable correction, most sociodemographic variables did not retain independent associations with risk status, indicating that age, marital status, occupation, and residence largely acted through clinical and behavioral mediators. Monthly income remained an exception: individuals in the 20,000–50,000 PKR bracket showed higher odds of being lower-risk than those earning < 20,000 PKR, while those earning > 100,000 PKR were more likely to be high-risk. Such non-linear socioeconomic effects are consistent with literature showing that financial capacity, healthcare-seeking behavior, medication adherence, and diagnostic access can vary significantly across socioeconomic gradients in South Asian countries [24,25].

Clinical comorbidities demonstrated the strongest and most consistent associations with fracture risk status. Diabetes was the most prominent predictor, with non-diabetic individuals exhibiting markedly lower odds of being in the high-risk group. This aligns with extensive evidence showing that type 2 diabetes substantially increases fracture risk despite normal or higher areal BMD, known as the “fracture–BMD paradox” [26]. Mechanisms underlying this paradox include accumulation of advanced glycation end products in collagen, reduced bone turnover, microangiopathy, and deterioration of both trabecular and cortical microarchitecture [27]. These biological pathways collectively weaken bone quality and plausibly explain the strong association observed in our cohort.

Short-term glucocorticoid exposure also emerged as a significant correlate of high-risk status. This finding is consistent with the 2022 American College of Rheumatology guideline, which classifies glucocorticoid exposure especially at moderate to high doses as a major criterion for early osteoporosis evaluation and pharmacologic intervention [28]. Glucocorticoids impair osteoblast differentiation, increase osteoclast survival, and reduce intestinal calcium absorption, leading to rapid bone loss and elevated fracture susceptibility [29]. The significance of short-term exposure in our model may reflect dose clustering, concurrent inflammatory disease, and interaction with other risk factors such as rheumatoid arthritis.

Smoking status demonstrated similarly robust associations: both current and former smokers had significantly elevated odds of high-risk status. This is consistent with systematic reviews showing approximately 30–40% increased hip fracture risk among smokers, driven by oxidative stress, direct osteotoxic effects, impaired calcium absorption, and hormonal alterations [30]. These metabolic effects underscore the potential value of pharmacist-delivered smoking cessation counseling as an osteoporosis risk-reduction strategy.

Environmental exposures also contributed meaningfully to risk stratification. Lack of sunlight exposure was strongly associated with high-risk status, reflecting the central role of cutaneous UVB exposure in maintaining adequate vitamin D levels particularly relevant in Pakistan, where vitamin D deficiency is highly prevalent [19]. Vitamin D insufficiency demonstrated a borderline association in the adjusted model. Evidence remains mixed: while large randomized trials such as VITAL in vitamin-D-replete populations showed no reduction in fractures with supplementation [31], multiple meta-analyses demonstrate that vitamin D (often 800–1000 IU/day) lowers fracture and fall risk in deficient or high-risk populations [32]. Given that deficiency rates in Pakistan routinely exceed 60% [19,30], our findings are directionally consistent with the broader evidence that targeted vitamin D correction may be more effective than population-wide supplementation strategies.

Rheumatoid arthritis and chronic kidney disease showed trends toward increased high-risk status but did not achieve statistical significance in the adjusted analysis. Nonetheless, these directions are biologically credible: CKD causes mineral metabolism abnormalities, secondary hyperparathyroidism, and cortical bone loss as components of CKD–MBD [33], while systemic inflammation, reduced mobility, elevated RANKL activity, and frequent use of glucocorticoids contribute to greater fracture risk in RA [34]. The absence of statistical significance in our sample may be due to small subgroup sizes or shared mechanisms involving glucocorticoid exposure and vitamin D levels.

Importantly, once clinical and behavioral variables were included in the model, most sociodemographic effects were attenuated or eliminated, suggesting that comorbidity burden, treatment exposures, and lifestyle patterns not demographic characteristics drive much of the variability in fracture-related risk status. This finding highlights the value of community pharmacies as strategic sites for early detection of high-risk individuals. Community pharmacists in Pakistan already play a growing role in chronic disease management, education, and screening programs [22,23], making integration of osteoporosis risk assessment a feasible next step.

Collectively, the results support a pharmacy-anchored approach to osteoporosis risk management that includes (i) opportunistic identification of individuals with diabetes, glucocorticoid exposure, smoking, or limited sunlight exposure; (ii) focused counseling on smoking cessation, vitamin D sufficiency, and lifestyle behaviors such as weight-bearing exercise; (iii) prioritized referrals for BMD testing in individuals with high-risk comorbidities; and (iv) collaborative management with prescribers to ensure guideline-concordant pharmacotherapy. Although these findings align with established mechanistic and observational evidence across multiple domains [26,27,28,29,30], the cross-sectional design precludes causal inference. Long-term studies and intervention trials within pharmacy settings are required to assess how effective these screening and counseling approaches are in real-world practice.

## 6. Strengths and Limitations

This study has several strengths. It evaluates osteoporosis-related risk factors directly within community pharmacy settings, a real-world environment where early identification is feasible. A full psychometric validation (e.g., reliability testing, CTT/IRT analyses) of the questionnaire was not conducted, which may limit the robustness of measurement properties; future studies should undertake comprehensive validation. The study incorporates a range of clinical, behavioral, and environmental predictors, providing a comprehensive socioecological assessment. However, limitations must be acknowledged. The cross-sectional design prevents causal inference. Convenience sampling may limit generalizability to the broader Pakistani population. Missing laboratory data (e.g., vitamin D and BMD) required exclusion from adjusted analyses. Self-reported behaviors may carry recall bias. The use of convenience sampling limits the generalizability of the findings to the wider Pakistani population, as participants may not represent all demographic or clinical subgroups. Additionally, although interviews were conducted by trained researchers using standardized protocols, the involvement of multiple interviewers may have introduced minor variability in data collection, and some residual interviewer bias cannot be entirely excluded.

## 7. Conclusions

This study highlights key clinical and behavioral determinants associated with fracture risk status among adults screened in community pharmacy settings in Lahore. Diabetes, glucocorticoid exposure, smoking, and inadequate sunlight or vitamin D intake emerged as important correlates of higher fracture risk, whereas adequate vitamin D status and regular sunlight exposure were associated with lower risk. Sociodemographic characteristics showed minimal independent influence after adjustment, underscoring that fracture risk in this population is driven primarily by modifiable clinical and lifestyle factors. These findings support the potential role of community pharmacists in early case finding, patient education, and referral for bone health assessment, particularly among individuals with diabetes, those receiving glucocorticoids, or those with limited sunlight exposure. While the cross-sectional design limits causal inference, the results demonstrate that pharmacy-based screening can identify high-risk groups who may benefit from targeted preventive strategies. Future research using longitudinal designs is recommended to evaluate the impact of pharmacist-led interventions on fracture outcomes and treatment adherence.

## Figures and Tables

**Table 1:** Demographic details of study population (n = 286).

Demographics	N (%)
**Gender**	
Male	105 (36.7)
Female	181 (63.3)
**Age groups**	
18–40 years	74 (25.9)
41–65 years	108 (37.8)
More than 65 years	104 (36.4)
**Occupation Type**	
Sedentary	79 (27.6)
Active	51 (17.8)
Unemployed	51 (17.8)
Retired	105 (36.7)
**Education Level**	
No Formal	101 (35.5)
Primary	99 (34.6)
Secondary	72 (25.2)
Graduate	7 (2.4)
Postgraduate	7 (2.4)
**Marital Status**	
Single	128 (44.8)
Married	74 (25.9)
Divorced	80 (28.0)
Widowed	4 (1.4)
**Residence**	
Urban	146 (51.0)
Rural	140 (49.0)
**Living Conditions**	
Own House	121 (42.3)
Rented	165 (57.7)
**Family Conditions**	
Joint Family	177 (61.9)
Living Alone/No family members	109 (38.1)
**Monthly Income**	
<20,000	52 (18.2)
20k–50k	97 (33.9)
50k–100k	75 (26.2)
>100k	62 (21.7)
**Menopausal status (for women)**	
Yes	156 (54.5)
No	130 (45.5)
**History of Fracture**	
Yes	133 (46.5)
No	153 (53.5)
**Chronic Kidney Disease (CKD)**	
Yes	108 (37.8)
No	178 (62.2)
**Diabetes**	
Yes	149 (52.1)
No	137 (47.9)
**Rheumatoid Arthritis**	
Yes	66 (23.1)
No	142 (49.7)
Not tested	78 (27.3)
**Family History of osteoporosis**	
Yes	114 (39.9)
No	172 (60.1)
**Use of glucocorticoids**	
Never	155 (54.2)
Short-term use	70 (24.5)
Long-term use	61 (21.3)
**Vitamin D level**	
Insufficient (20–29 ng/mL)	59 (20.6)
Sufficient (≥30 ng/mL)	150 (52.4)
Not tested	77 (26.9)
**Bone mineral density**	
Normal	139 (48.6)
Osteopenia	33 (11.5)
Osteoporosis	79 (27.6)
Not tested	35 (12.2)
**Smoking Status**	
Never	108 (37.8)
Current	115 (40.2)
Ex Smoker	63 (22.0)
**Physical Activity (≥150 min/week?)**	
Yes	161 (56.3)
No	125 (43.7)
**Vitamin D & Calcium diet intake**	
Low intake	80 (28.0)
Moderate intake	18 (6.3)
Adequate intake	149 (52.1)
Don’t know	39 (13.6)
**Alcohol consumption**	
Yes	40 (14.0)
No	246 (86.0)
**Sunlight exposure**	
Yes	175 (61.2)
No	111 (38.8)
**Body Mass Index BMI**	
Underweight/Normal	108 (37.8)
Overweight	112 (39.2)
Obese	66 (23.1)

**Table 2:** Socio-Demographic Predictors Influencing Osteoporosis Control: Findings from a Cross-Sectional Study Conducted in Community Pharmacies of Lahore, Pakistan (n = 286).

Variables	Fracture Risk Status (N%)		Univariate Analysis		Multivariate Analysis	
	Lower-Risk Group	High-Risk Group	Crude OR (95% CI)	*p*-Value	Adjusted OR (95% CI)	*p*-Value
**Gender**						
Female	83 (45.9)	98 (54.1)	Referent		Referent	
Male	48 (45.7)	57 (54.3)	0.62 (0.38–1.01)	0.056	0.64 (0.37–1.10)	0.104
**Age groups**						
18–40 years	44 (59.5)	30 (40.5)	Referent		Referent	
41–65 years	62 (57.4)	46 (42.6)	0.92 (0.50–1.68)	0.783	1.08 (0.56–2.10)	0.810
More than 65 years	46 (44.2)	58 (55.8)	0.54 (0.30–0.99)	0.046	0.59 (0.30–1.15)	0.120
**Occupation Type**						
Sedentary	44 (55.7)	35 (44.3)	Referent		Referent	
Active	23 (45.1)	28 (54.9)	0.65 (0.32–1.33)	0.239	0.85 (0.38–1.89)	0.694
Unemployed	27 (52.9)	24 (47.1)	0.89 (0.44–1.81)	0.758	0.87 (0.39–1.93)	0.731
Retired	58 (55.2)	47 (44.8)	0.98 (0.55–1.77)	0.951	1.03 (0.53–2.00)	0.923
**Education Level**						
No formal	59 (58.4)	42 (41.6)	Referent		Referent	
Primary	45 (45.5)	54 (54.5)	0.59 (0.34–1.04)	0.067	0.63 (0.34–1.16)	0.139
Secondary	39 (54.2)	33 (45.8)	0.84 (0.46–1.55)	0.578	0.85 (0.43–1.68)	0.638
Graduate	3 (42.9)	4 (57.1)	0.53 (0.11–2.51)	0.427	0.62 (0.12–3.23)	0.575
Postgraduate	6 (85.7)	1 (14.3)	4.27 (0.50–36.80)	0.186	3.81 (0.40–36.11)	0.244
**Marital Status**						
Single	69 (53.9)	59 (46.1)	Referent		Referent	
Married	42 (56.8)	32 (43.2)	1.45 (0.77–2.74)	0.251	1.40 (0.71–2.76)	0.338
Divorced	38 (47.5)	42 (52.5)	1.29 (0.74–2.26)	0.369	1.43 (0.72–2.85)	0.308
Widowed	3 (75.0)	1 (25.0)	3.32 (0.33–33.25)	0.308	3.35 (0.31–36.29)	0.321
**Living Conditions**						
Own House	63 (52.1)	58 (47.9)	Referent		Referent	
Rented	89 (53.9)	76 (46.1)	0.93 (0.58–1.48)	0.754	1.26 (0.56–2.81)	0.576
**Family Condition**						
Joint Family	92 (52.0)	85 (48.0)	Referent		Referent	
Alone	60 (55.0)	49 (45.0)	0.96 (0.59–1.76)	0.351	1.09 (0.41–2.99)	0.576
**Residence**						
Urban	73 (50.0)	73 (50.0)	Referent		Referent	
Rural	79 (56.4)	61 (43.6)	0.77 (0.48–1.23)	0.276	0.62 (0.28–1.38)	0.241
**Monthly Income (PKR)**						
<20,000	41 (78.8)	11 (21.2)	Referent		Referent	
20k–50k	49 (50.5)	48 (49.5)	3.65 (1.68–7.93)	0.001	2.98 (1.27–7.03)	0.012
50k–100k	43 (57.3)	32 (42.7)	1.32 (0.72–2.41)	0.374	1.15 (0.59–2.23)	0.682
>100k	19 (30.6)	43 (69.4)	0.43 (0.22–0.85)	0.014	0.37 (0.17–0.80)	0.012

**Referent:** “Referent” indicates the reference category used for comparison in the logistic regression models. All odds ratios (OR) for other categories are calculated relative to this baseline group. **Significance criteria:** *p* < 0.05 was considered statistically significant. **Crude Odds Ratios (OR):** Define the crude OR as unadjusted estimates representing the direct relationship between each predictor and the outcome, without controlling for other variables. **Adjusted Odds Ratios (AOR):** Specify that the AOR accounts for potential confounders by adjusting for covariates included in the model, providing a more accurate estimate of the relationship. Effect magnitudes were interpreted using adjusted odds ratios (AORs) and their 95% confidence intervals, which are the standard effect size indicators for logistic regression models. No ANOVA-type effect size measures were used.

**Table 3:** Clinical and Environmental Predictors Influencing Osteoporosis Control: Findings from a Cross-Sectional Study Conducted in Community Pharmacies of Lahore, Pakistan (n = 286).

Variables	Lower-Risk Group n (%)	High-Risk Group n (%)	Crude OR (95% CI)	*p*-Value	Adjusted OR (95% CI)	*p*-Value
**Diabetes**						
Yes	23 (15.4)	126 (84.6)	Referent	—	Referent	—
No	129 (94.2)	8 (5.8)	0.006 (0.003–0.013)	<0.001	0.005 (0.0007–0.040)	<0.001
**Glucocorticoid Use**						
Never	130 (83.9)	25 (16.1)	Referent	—	Referent	—
Short-term	16 (22.9)	54 (77.1)	2.72 (0.99–7.46)	0.053	32.33 (3.05–342.66)	0.004
Long-term	6 (9.8)	55 (90.2)	47.67 (18.52–122.66)	<0.001	12.62 (0.48–330.83)	0.128
**Smoking Status**						
Never	81 (75.0)	27 (25.0)	Referent	—	Referent	—
Current	37 (32.2)	78 (67.8)	0.16 (0.09–0.28)	<0.001	14.23 (2.66–76.04)	0.002
Ex-smoker	34 (54.0)	29 (46.0)	0.39 (0.20–0.76)	0.005	4.95 (1.06–23.11)	0.042
**Sunlight Exposure**						
Yes	109 (62.3)	66 (37.7)	Referent	—	Referent	—
No	43 (38.7)	68 (61.3)	0.38 (0.23–0.62)	<0.001	7.09 (1.38–36.40)	0.019
**Chronic Kidney Disease**						
Yes	27 (25.0)	81 (75.0)	Referent	—	Referent	—
No	125 (70.2)	53 (29.8)	7.08 (4.12–12.16)	<0.001	0.82 (0.13–5.27)	0.833
**Rheumatoid Arthritis**						
Yes	26 (39.4)	40 (60.6)	Referent	—	Referent	—
Group 2	103 (72.5)	39 (27.5)	4.06 (2.19–7.52)	<0.001	0.25 (0.06–1.05)	0.059
Group 3	23 (29.5)	55 (70.5)	0.64 (0.32–1.29)	0.212	0.92 (0.27–3.12)	0.888
**Vitamin D Level**						
Sufficient	111 (74.0)	39 (27.0)	Referent	—	Referent	—
Insufficient	31 (52.5)	28 (47.5)	34.61 (16.58–132.61)	<0.001	0.093 (0.008–1.07)	0.057

**Referent:** Reference category used for comparison in logistic regression and chi-square analyses. **Significance criteria:** *p* < 0.05 was considered statistically significant.

## Data Availability

The original contributions presented in this study are included in the article. Further inquiries can be directed to the corresponding author.

## Acronyms

The following abbreviations are used in this manuscript:

BMD	Bone Mineral Density
CKD	Chronic Kidney Disease
RA	Rheumatoid Arthritis
OR	Odds Ratio
AOR	Adjusted Odds Ratio
CI	Confidence Interval
BMI	Body Mass Index
Vit D	Vitamin D
FR	Fracture Risk
SPSS	Statistical Package for the Social Sciences
